# A case of annular pancreas complicated with pancreatic ductal adenocarcinoma diagnosed with linear endoscopic ultrasound

**DOI:** 10.1055/a-2603-7352

**Published:** 2025-06-18

**Authors:** Songming Ding, Hengkai Zhu, Xuliang Chen, Shanjie Dong, Qiyong Li, Hua Guo

**Affiliations:** 1 636046 Division of Hepatobiliary and Pancreatic Surgery, Shulan (Hangzhou) Hospital Affiliated to Zhejiang Shuren University, Shulan International Medical College, Hangzhou, China

Annular pancreas (AP) is an uncommon congenital anomaly characterized by pancreatic tissue partially or completely encircling the duodenum 1 . AP complicated with pancreatic ductal adenocarcinoma (PDAC) is extremely rare to be reported 2 3 . The diagnosis of AP can be confirmed by endoscopic ultrasound (EUS) 4 5 . Here, we present a patient diagnosed with AP accompanied by PDAC through linear EUS and EUS-guided fine-needle biopsy (EUS-FNB).

The patient was a 78-year-old woman suffering from abdominal distension and anorexia, which had lasted for 2 months. In the last 1 week, the above-mentioned malaise was intensified, and abdominal pain occurred occasionally. Thus, she was admitted to our hospital in order to seek clinical evaluation and treatment in March 2023.

On admission, physical examination revealed no anemia and yellowish discoloration of skin and sclera. Carbohydrate antigen 19-9 (CA19-9) was elevated to 477.5 U/ml (≤43 U/ml). Contrast-enhanced computed tomography (CT) suggested a ring of pancreas surrounding the duodenum and a space-occupying lesion in the pancreatic head ( Fig. 1 ). Contrast-enhanced magnetic resonance imaging (MRI) indicated the existence of AP, but it was not sure whether it was combined with focal pancreatitis or pancreatic cancer ( Fig. 2 ). Linear EUS showed that the pancreas extended to the outer side of the duodenum ( Fig. 3 **a** ). Meanwhile, the pancreatic duct was observed coursing around the duodenum ( Video 1 ). AP was confirmed. Furthermore, linear EUS showed a hypoechoic mass in the intersection of AP and pancreatic head, measuring 24 × 21 mm, which resulted in significant dilation of the accessory pancreatic duct ( Fig. 3 **b** ). Noteworthily, the descending part of the duodenum had a narrowed lumen and the endoscopy could not pass through ( Fig. 4 ). Using a 22-gauge needle, we punctured the hypoechoic mass at the duodenal bulb. Histopathology showed poorly differentiated PDAC ( Fig. 5 ).

**Fig. 1:**
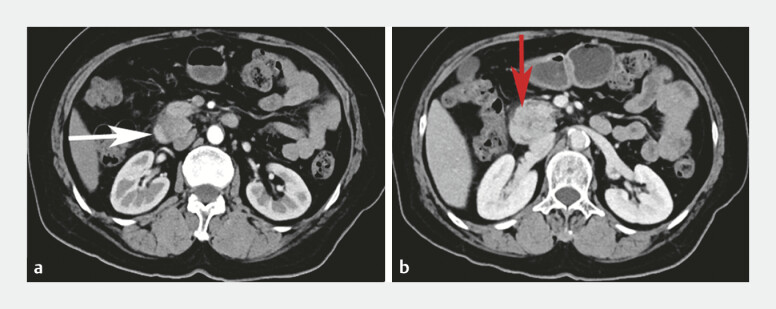
Contrast-enhanced abdominal CT scan. **a** A ring of pancreas was seen around the second part of the duodenum (white arrow). **b** A hypodense mass was indicated (red arrow). Abbreviation: CT, computed tomography.

**Fig. 2:**
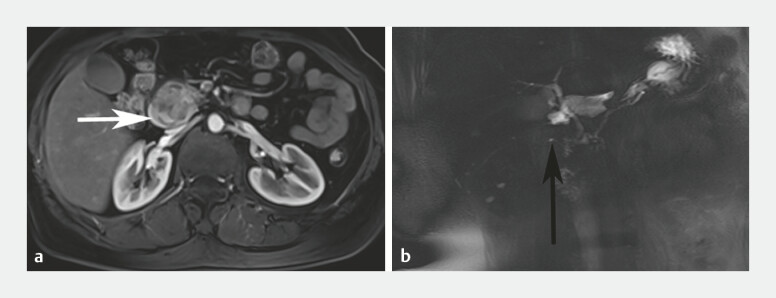
Contrast-enhanced abdominal MRI. **a** MRI showed a ring of pancreas surrounding the descending part of the duodenum (white arrow). **b** MRI depicted the course of the annular pancreatic duct (black arrow). Abbreviation: MRI, magnetic resonance imaging.

**Fig. 3:**
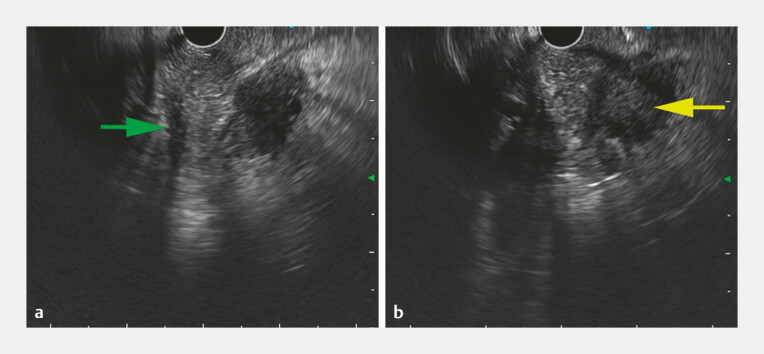
Linear EUS view at the duodenal bulb. **a** Pancreatic parenchyma extension to the outer side of the duodenum with the hypoechoic pancreatic duct coursing around the duodenum (green arrow). **b** EUS represented a hypoechoic lesion in the intersection of the annular pancreas (AP) and pancreatic head (yellow arrow). Abbreviation: EUS, endoscopic ultrasound.

Linear endoscopic ultrasound (EUS) scanning and EUS-guided fine-needle biopsy (EUS-FNB).Video 1

**Fig. 4:**
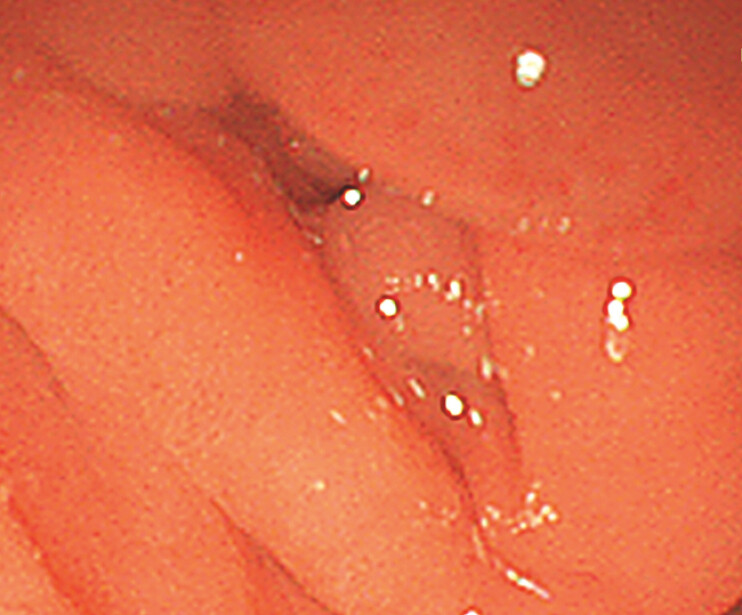
The endoscopy showed the narrowing of the second part of the duodenum.

**Fig. 5:**
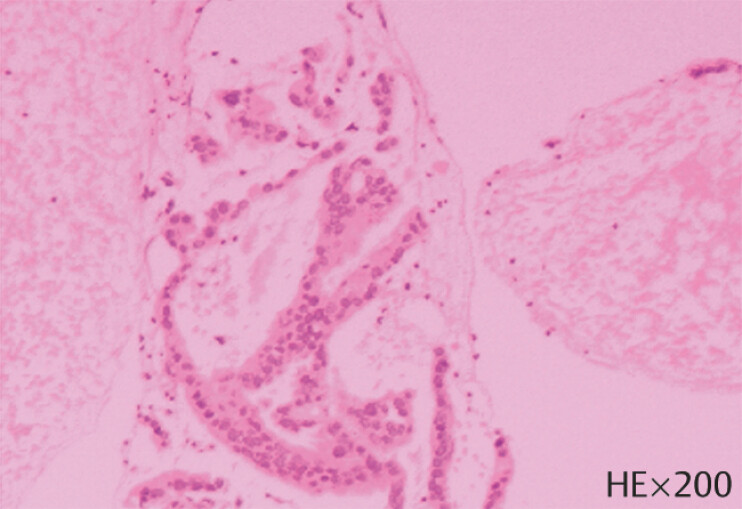
A pathological examination of the EUS-FNB showed the poorly differentiated adenocarcinoma. Abbreviation: EUS-FNB, endoscopic ultrasound-guided fine-needle biopsy.

In conclusion, EUS is effective for the definitive diagnosis of AP. If CA19-9 is elevated in AP patients, the possibility of coexisting PDAC should be considered.

Endoscopy_UCTN_Code_CCL_1AZ_2AL

## Notes

Endoscopy E-Videos https://eref.thieme.de/e-videos
*E-Videos* is an open access online section of the journal *Endoscopy* , reporting on interesting cases and new techniques in gastroenterological endoscopy. All papers include a high-quality video and are published with a Creative Commons CC-BY license. Endoscopy E-Videos qualify for HINARI discounts and waivers and eligibility is automatically checked during the submission process. We grant 100% waivers to articles whose corresponding authors are based in Group A countries and 50% waivers to those who are based in Group B countries as classified by Research4Life (see: https://www.research4life.org/access/eligibility/ ). This section has its own submission website at https://mc.manuscriptcentral.com/e-videos .
